# Immune Response and Modeled Duration of Protection Following a Single 60 μg Hepatitis B Vaccine Booster in Susceptible Chinese University Students

**DOI:** 10.3390/vaccines14040345

**Published:** 2026-04-14

**Authors:** Xianwei Luo, Wenxiang Zhou, Shujie Zhou, Feiyang Song, Shouyan Geng, Haiyang Xu, Yuhui Cheng, Mingxue Ren, Yan Dong, Ling Lin, Leijing Mao, Binbing Wang, Yu Chai, Pengcheng Miao, Shaodi Ma, Jihai Tang

**Affiliations:** 1 Anhui Provincial Center for Disease Control and Prevention, Hefei 230601, China; lxw@ahcdc.com.cn (X.L.); zsj@ahcdc.com.cn (S.Z.); sfy@ahcdc.com.cn (F.S.); rmx@ahcdc.com.cn (M.R.); dy@ahcdc.com.cn (Y.D.); ll@ahcdc.com.cn (L.L.); maolj@ahcdc.com.cn (L.M.); wbb@ahcdc.com.cn (B.W.); chaiyu@ahcdc.com.cn (Y.C.); mpch@ahcdc.com (P.M.); msd@ahcdc.com.cn (S.M.); 2 School of Public Health, Bengbu Medical University, Bengbu 233030, China; zwxbbmu@stu.bbmu.edu.cn; 3 Chuzhou Center for Disease Control and Prevention, Chuzhou 239000, China; 18019819083@163.com; 4 Fuyang Center for Disease Control and Prevention, Fuyang 236000, China; haiyang86xu@163.com; 5 Xuancheng Center for Disease Control and Prevention, Xuancheng 242000, China; hui7928@163.com

**Keywords:** hepatitis B vaccine, mathematical modeling, immunogenicity, university students

## Abstract

Background: Since China incorporated the hepatitis B vaccine into its Expanded Program on Immunization (EPI) in 2002, the first cohort of infants to receive the full vaccination series has now reached college age. As vaccine-induced antibodies gradually wane, this cohort faces a higher risk of infection. Therefore, we assessed their current seroprotection status and evaluated the immunogenicity and short-term antibody kinetics of a single 60 μg booster dose in susceptible individuals, while also constructing a model of expected duration of protection. Methods: In a multicenter study across three Anhui universities, 2988 students were screened for HBV markers. Among them, 160 who tested negative for all five markers received a single 60 μg booster. Antibody titers were monitored for 1–5 months. Results: Serological screening showed 0.33% HBsAg positivity, 36.28% anti-HBs positivity, and 63.02% negativity for all markers, indicating high susceptibility. After the booster, seroprotection rate (SPR) remained >85% throughout follow-up, and anti-HBs geometric mean concentration (GMC) peaked at 1–2 months. Stratified analysis based on immune response status revealed that the proportion of high responders (≥100 mIU/mL) peaked early and then gradually declined, whereas the proportion of low responders (10–99.99 mIU/mL) increased over the follow-up period. A linear mixed-effects model predicted that protective levels (anti-HBs ≥10 mIU/mL) would persist for an average of 32.8 months. Conclusions: A substantial proportion of university students lack protective immunity against hepatitis B. A single 60 μg booster rapidly and effectively induced protection, demonstrating strong immunogenicity. These findings support implementing efficient booster strategies in university settings.

## 1. Introduction

According to the World Health Organization (WHO), approximately 254 million people worldwide were living with chronic hepatitis B as of 2022, with the highest burden concentrated in the Western Pacific, South-East Asia, and African regions [1,2]. Hepatitis B virus (HBV) infection represents a significant global public health challenge, imposing a substantial disease burden worldwide. The hepatitis B vaccine stands as the most effective tool for preventing hepatitis B. By the end of 2019, 189 countries and regions had incorporated the hepatitis B vaccine into their national immunization programs. Global coverage of the three-dose hepatitis B vaccine series for infants increased from 1% in 1990 to 85% in 2019 [3].

In China, approximately 75 million individuals are chronically infected with hepatitis B [4]. China incorporated the hepatitis B vaccine into the Expanded Program on Immunization (EPI) in 2002, providing free vaccination for all newborns. By 2005, the hepatitis B vaccine had become entirely free of charge [5]. Recent surveys indicate that hepatitis B vaccine coverage among Chinese newborns has exceeded 95% [6]. The first cohort of newborns immunized under this policy is now entering university. However, vaccine-induced antibody titers naturally decline over time. University students, due to multiple high-risk behaviors such as tattooing, unprotected sexual activity, ear piercing, and accidental blood exposure, coupled with low awareness of hepatitis B-related knowledge, face potential HBV exposure risks upon entering densely populated, socially active university environments. Consequently, they constitute a susceptible population for hepatitis B infection.

Although the majority of current university students received the hepatitis B vaccine during infancy, the long-term persistence of vaccine-induced protective antibodies remains debated. Evidence on the duration of protection is inconsistent. One study reported that approximately 75% of individuals maintained protective antibody titers 17–20 years after receiving the hepatitis B vaccine at birth [7]. Another study, however, found that only 41.8% of adolescents remained seroprotected (anti-HBs ≥ 10 mIU/mL) 15–17 years after completing the primary infant vaccination series [8]. Furthermore, a meta-analysis revealed that approximately 40% of individuals experience a decline in antibody titers below the protective threshold within 10 years post-vaccination [9]. Currently, there is a lack of large-scale serological surveys targeting university students born after 2002 who have now reached adulthood, leaving their immune protection status during university years to be assessed.

According to the WHO, hepatitis B vaccination is recommended for young adolescents, household and sexual contacts of HBsAg-positive persons, and anyone else at risk of HBV infection [10]. In China, however, adult hepatitis B vaccination remains voluntary and out-of-pocket. The standard adult schedule consists of a three-dose (20 μg) regimen administered at 0, 1, and 6 months [11]. Although this regimen demonstrates good immunogenicity and safety, its complexity and lengthy intervals often result in poor compliance, with studies reporting completion rates below 50% [12,13]. Consequently, enhancing compliance and exploring high-dose, short-course immunization strategies have become research priorities. As a high-dose booster strategy, the 60 μg hepatitis B vaccine has the potential to improve compliance and coverage. However, whether this formulation exhibits distinct immunogenicity profiles compared to conventional regimens warrants further investigation. Globally, real-world evidence on the use of the 60 μg hepatitis B vaccine for accelerated adult immunization remains limited. In particular, large-scale studies evaluating its safety and post-vaccination antibody titer dynamics are scarce.

This study aims to assess the seroprotection status of university students to determine the need for on-campus hepatitis B booster immunization. Concurrently, susceptible individuals will receive a single 60 μg booster dose, with short-term follow-up to assess the immunogenicity, including the induction of rapid seroprotection and post-vaccination antibody kinetics. Given the low compliance and high dropout rates associated with the standard three-dose vaccination regimen among mobile populations such as college students, designing a trial to compare this regimen with the standard one poses significant practical challenges. Therefore, we investigated whether a simplified regimen consisting of a single 60 μg hepatitis B vaccine could successfully and rapidly induce an immune response. The findings will provide preliminary insights into the optimal timing for subsequent booster doses and offer scientific evidence to inform hepatitis B immunization strategies in university populations.

## 2. Material and Methods

### 2.1. Study Design

**Figure 1:**
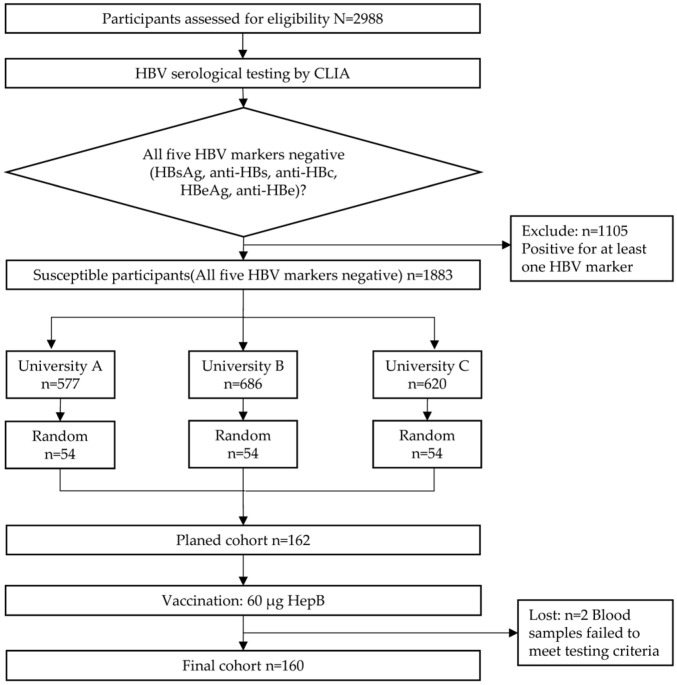
The flow diagram of participants through the study.

This study constitutes a two-stage investigation combining cross-sectional screening with prospective intervention, conducted across three higher education institutions in Anhui Province, China. The first stage employed a serological cross-sectional survey to assess hepatitis B immunization status among university students. The second stage recruited participants identified as susceptible from the screening results, administering a single 60 μg dose of recombinant hepatitis B vaccine (Saccharomyces cerevisiae; Shenzhen Kangtai Biological Products Co., Ltd., Shenzhen, China), followed by short-term follow-up to evaluate immunological response and antibody dynamics (Figure 1).

Participants were university students aged ≥16 years with a history of hepatitis B vaccine. Venous blood samples were collected and analyzed using chemiluminescence immunoassay to detect five serological markers: hepatitis B surface antigen (HBsAg), antibody against hepatitis B surface antigen (anti-HBs), antibody against hepatitis B core antigen (anti-HBc), hepatitis B e antigen (HBeAg), and antibody against hepatitis B e antigen (anti-HBe). Susceptible individuals were defined as those negative for all five markers. Participants at each university were randomly selected using simple random sampling based on computer-generated random numbers. This study was designed to assess the short-term antibody kinetics following a single 60 μg hepatitis B vaccine booster and to acquire early-phase data for modeling long-term protection. A traditional balanced follow-up schedule—in which all participants are assessed at identical time points—may not optimally capture the detailed trajectory of antibody dynamics in the initial months. Therefore, we implemented an unbalanced longitudinal design by assigning different final follow-up time points (Month 3, Month 4, and Month 5) across the three participating universities (University A, University B, and University C) to explore the pattern of antibody changes within the first five months after vaccination. Inclusion and exclusion criteria were as follows:

Inclusion criteria:

(1) University students aged 16 years or older;
(2) Healthy individuals with a history of hepatitis B vaccine;
(3) Obtained signed informed consent from the subject or their guardian.

Exclusion criteria:

(1) Individuals with fever or acute/chronic severe illness;
(2) Known allergy to any component of the 60 μg vaccine, including yeast cells or adjuvant ingredients;
(3) History of convulsions (personal or familial), epilepsy, or allergic constitution;
(4) Administration of live attenuated vaccines within 14 days or subunit/inactivated vaccines within 7 days before trial vaccine administration;
(5) Current participation in another clinical trial;
(6) Participants deemed unsuitable by the investigator.

### 2.2. Sample Size Calculation

To ensure the study’s efficacy and reliability, this research first recruited 2988 university students to complete baseline serological screening in accordance with vaccine safety monitoring requirements. Building upon this, and based on prior studies indicating a 90% rapid surface antibody positivity rate following adult hepatitis B vaccine, the sample size was calculated using the formula $N=\;Z_{\alpha /2}^{2}p(1-p)/\delta^{2}$. Assuming *α* = 0.05 and a margin of error of *δ* = 0.05, the required sample size was determined to be 139 individuals. Accounting for a 10% dropout rate, each group required monitoring of 155 individuals. To ensure balanced sample distribution across universities, 54 participants were recruited from each of the three institutions, totaling 162 individuals for follow-up. Two blood samples failed to meet measurement standards, resulting in a final study cohort of 160 participants.

### 2.3. Lab Testing

Chemiluminescence immunoassay was employed to determine HBsAg, anti-HBs, anti-HBc, HBeAg, and anti-HBe. Testing was performed using a fully automated chemiluminescent immunoassay analyzer (Medicalsystem Biotechnology Co., Ltd., Ningbo, China, model MS-I2280) with manufacturer-supplied reagent kits. Results were interpreted according to the reagent manual. Positive criteria were defined as follows: HBsAg concentration ≥ 0.05 IU/mL, anti-HBs concentration ≥ 10 mIU/mL, which according to the WHO position paper is considered a reliable marker of seroprotection when measured 1–3 months post-vaccination [14], HBeAg absorbance ratio ≥ 1, anti-HBe absorbance ratio ≥ 1, and anti-HBc absorbance value to critical value ratio ≥ 1. Conversely, results were deemed negative. Suspicious initial test results required repeat testing. For the samples with questionable initial test results, we conducted repeated tests using the same serum samples for storage purposes to confirm the results.

### 2.4. Mathematical Modeling for Long-Term Anti-HBs Antibody Duration

To predict antibody persistence following single 60 μg hepatitis B vaccine, we constructed a linear mixed-effects model based on longitudinal follow-up data. This model simultaneously accounts for the correlation of repeated measurements, inter-individual heterogeneity, and measurement error, making it suitable for the non-balanced follow-up design in this study. The model comprises four components: fixed-effect parameters, random effects, serial correlation, and measurement error. Time (months), university, age, and gender were incorporated as fixed effects in the analysis, with measurement error variance permitted to vary over time.

### 2.5. Data Analysis

A database was established using Epidata 3.1, and data were processed via the open-source package lme4 (www.r-project.org) in R 4.4.1. A linear mixed-effects model was employed to predict seropositivity rates following booster vaccination (with an anti-HBs threshold of 10 mIU/mL). Antibody titers were log-transformed to observe mean trends. The model utilized all available data during follow-up periods. Model fit was assessed using AIC and BIC, alongside ICC and mixed-effects model coefficient of determination (R^2^).

## 3. Results

### 3.1. Seroprevalence of HBV Markers Among Screened Students

Analysis of specific markers showed that the positivity rates for HBsAg, anti-HBs, anti-HBc, HBeAg, and anti-HBe were 0.33% (10/2988), 36.28% (1084/2988), 1.07% (32/2988), 0.10% (3/2988), and 0.30% (9/2988). Serological screening of 2988 eligible participants revealed 11 distinct serological profiles. A total of 1883 (63.02%) participants tested negative for all five HBV markers, indicating susceptibility to infection. Among the 1071 participants who were positive only for anti-HBs, in the HBsAg-positive cases, three were simultaneously positive for anti-HBc and HBeAg; four were positive for anti-HBc and anti-HBe; and three were positive only for anti-HBc (Table 1).

### 3.2. Baseline Characteristics of Participants

According to the inclusion and exclusion criteria, this study analyzed 160 participants who signed informed consent forms and received a single 60 μg dose of hepatitis B vaccine. The mean age of the participants was 18.98 ± 0.83 years, comprising 61 males and 99 females; there were 52 students from University A, and 54 students each from University B and C (Table 2).

### 3.3. Immunogenicity in Participants

Following the administration of a single 60 μg booster dose, participants across all three universities mounted a robust and rapid immune response. As detailed in Table 2, the seroprotection rate (SPR) among students in University A was 98.08% (51/52; 95% CI: 89.74–99.95%) at Month 1, reaching 100% (52/52) at Month 2 and Month 3. In University B, the SPR was 87.04% (47/54), 92.59% (50/54), and 90.74% (49/54) at Month 1, Month 2 and Month 4, respectively. Similarly, in University C, the SPR was 85.19% (46/54), 92.59% (50/54), and 94.44% (51/54) at Month 1, Month 2 and Month 5. Notably, all groups maintained SPRs above 85% from the first month onward. The geometric mean concentration (GMC) of anti-HBs peaked at 1–2 months post-vaccination before gradually declining. In University A, the GMC was 386.87 mIU/mL at Month 1, increased to 475.05 mIU/mL at Month 2, and was 398.20 mIU/mL at Month 3. For University B, the GMC decreased from 428.98 mIU/mL (Month 1) to 373.94 mIU/mL (Month 2) and 280.01 mIU/mL (Month 4). University C showed a comparable trend, with GMCs of 417.86 mIU/mL, 396.61 mIU/mL, and 230.49 mIU/mL at Month 1, Month 2, and Month 5, respectively (Table 3).

### 3.4. Distribution of Anti-HBs Titers

**Figure 2:**
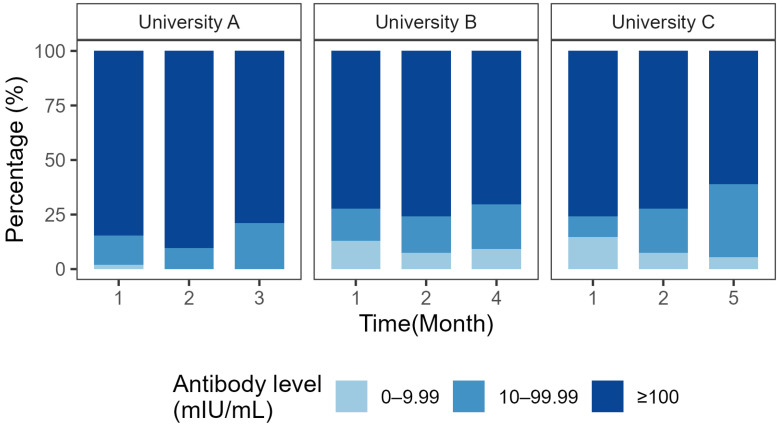
Distribution of hepatitis B antibody levels.

Anti-HBs titers were classified into three categories reflecting the participants’ immune response status: high responders (≥100 mIU/mL), low responders (10–99.99 mIU/mL), and non-responders (<10 mIU/mL). At Month 1, the proportion of high responders across the three universities ranged from 72% to 85%. Over the follow-up period, the proportion of high responders gradually declined, whereas the proportion of low responders increased correspondingly, rising from 9–15% at Month 1 to 20–33% at the final follow-up. The proportion of non-respondents remained low throughout the study, never exceeding 15% at any point in time, and did not exceed 10% at the final follow-up at all three universities (Figure 2).

### 3.5. Modeling Result

**Figure 3:**
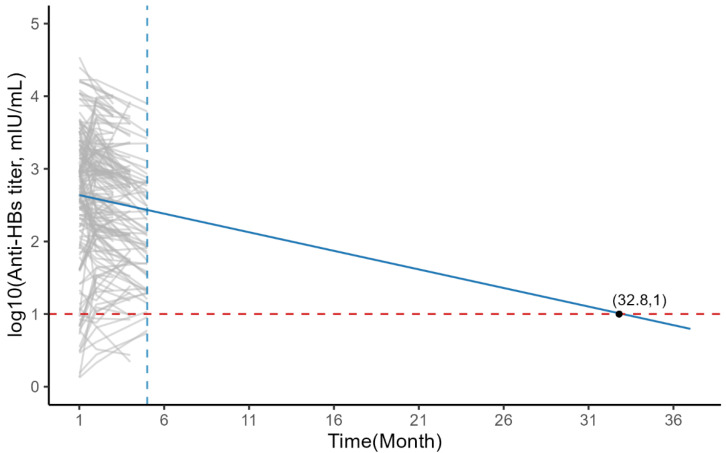
Observed individual profiles and population-averaged estimation of anti-HBs titers.

We included time (Month), university, age, and gender as fixed effects in a linear mixed-effects model, where only time (Month) has a significant impact and all other variables had no significant impact; after selecting by AIC and BIC, we finalized the equation of the fitted mixed-effects model as log10 (GMC)~time (Month) + (1 + time (Month)|id), thus fitting the variation in antibodies, and Table 4 shows the parameter estimates and standard errors; we fitted all the follow-up results into the same model, which utilized the observed data to predict the long-term persistence of antibodies. It was found that the average antibodies in the population decreased over time, with the average antibody level dropping below the protective antibody level at 32.8 months (Figure 3).

## 4. Discussion

Since the inclusion of the hepatitis B vaccine in China’s Expanded Program on Immunization (EPI) over two decades ago, the country has achieved remarkable success in pediatric infection control. Four national serosurveys have shown that the HBsAg positivity rate among children under five years of age has remained below 1% since 2006, demonstrating the effectiveness of the childhood hepatitis B vaccination strategy [15]. While the HBV infection rate among children has been effectively controlled, a national immunization program for adults has not been established; adult vaccination remains voluntary and self-paid. Previous surveys indicate that the HBV disease burden in the adult population remains substantial [4,16]. The university student cohort represents a crucial breakthrough point for adult hepatitis B prevention and control. Research into hepatitis B prevention measures for university students and exploration of optimal immunization strategies are of significant importance for reducing hepatitis B infection rates across the entire population, alleviating the burden on patients’ families, and maintaining social stability.

To investigate the current hepatitis B immunity status among university students, this study conducted a multicenter epidemiological survey within Anhui Province. The results revealed that the HBsAg positivity rate among university students was merely 0.33%, while the anti-HBs positivity rate stood at only 36.28%. A substantial proportion of 63.02% tested negative for all five hepatitis B markers, indicating that the vast majority of university students constitute a susceptible population for hepatitis B. This immunological profile of low infection rates coupled with high susceptibility aligns with findings from other universities [17,18,19], highlighting the latent risk of HBV transmission. This may stem from the natural waning of antibodies acquired through infant immunization over time, potentially rendering them insufficient for effective immune protection among the university cohort. Consequently, this underscores the necessity for booster immunization within this population.

To evaluate a practical booster strategy, we administered a single 60 μg dose of hepatitis B vaccine to susceptible university students and followed them for five months. Multiple studies have previously demonstrated that the 60 μg hepatitis B vaccine exhibits favorable immunogenicity and safety profiles [20,21]. The present study demonstrates that when university students received a single 60 μg hepatitis B vaccine, seroconversion rates exceeded 85% at all observation time points, reaching this level one month post-vaccination. This indicates rapid establishment of immune response, which showed comparable seroconversion rates one month after vaccination in healthy Chinese individuals aged 18–25 years [22]. These findings indicate that a single 60 μg booster dose can elicit an immune response in young adults that is comparable in magnitude and kinetics to that induced by more complex multi-dose regimens. This study further characterizes the antibody kinetics of the 60 μg hepatitis B vaccine, with the GMC peaking 1 to 2 months after vaccination. This aligns with prior research indicating that anti-HBs titers typically peak one month after completion of the full immunization course [23]. Antibody titers gradually declined after reaching their peak, with the rapid peak attainment confirming effective activation of immune memory. Notably, despite all enrolled students having baseline antibody titers below the protective threshold (<10 mIU/mL), the 60 μg hepatitis B vaccine elicited a robust immune response within a short timeframe. This suggests that immune memory cells established during infant vaccination persist for over a decade. Furthermore, the study observed a continuing upward trend in antibody seroconversion rates shortly after vaccination, further reflecting the sustained activation of the immune response. Previous studies in healthy adults have shown that a booster dose of the hepatitis B vaccine can effectively induce serum protection in individuals who did not respond to the first dose, and that a high-dose regimen provides better protection than a low-dose booster regimen [24]. Therefore, for the small number of non-responders identified, it is recommended that they receive a supplemental dose, and that follow-up studies with larger sample sizes and longer follow-up periods be conducted.

In the analysis of anti-HBs titer stratification, participants were classified according to their immune response status as high responders (≥100 mIU/mL), low responders (10–99.99 mIU/mL), and non-responders (<10 mIU/mL). The UK and Germany believe that when the anti-HBs level reaches above 100 mIU/mL, this figure can more reliably indicate the presence of a specific immune response and provide better immune protection [25]. At Month 1, the proportion of high responders across the three universities ranged from 72% to 85%, indicating that a single 60 μg booster dose rapidly elicits a robust immune response. Over the follow-up period, the proportion of high responders gradually declined, whereas the proportion of low responders increased correspondingly, suggesting that antibody levels in some vaccinees are progressively approaching the lower limit of the protective threshold, albeit without falling below it in the short term. These findings confirm the rapid immunogenicity of this booster regimen and underscore the importance of long-term monitoring of antibody persistence to inform the optimal timing of subsequent boosters.

In terms of model construction, Vellinga proposed a linear mixed-effects model comprising both fixed and random effects [26]. This model allows for the inclusion of time, university, age, and gender as fixed effects, while assigning random intercepts and slopes to each individual, thereby enabling a comprehensive decomposition of the variance in antibody changes. After log transformation, the overall mean anti-HBs titer exhibited an approximately linear downward trend over the observation period. Model results indicate that only time has a significant effect on antibody trajectories, while university, age, and gender all lack significant effects. Therefore, we adopted this model for the analysis of our dataset. In recent years, such linear mixed-effects models have been widely applied in numerous studies on antibody persistence [27,28,29]. Based on this model, we predict that the average duration of protective antibody levels (anti-HBs ≥ 10 mIU/mL) following a single 60 μg booster dose is 2–3 years.

In our model, no significant association was found between age or gender and antibody immunogenicity. However, previous studies have shown that vaccine efficacy is lower in individuals aged 40 years or older compared to younger adults, a finding primarily attributed to the gradual decline in immune system function with age [30]. Regarding gender, no significant gender differences were observed in this study, a result consistent with some of the literature [31]. However, some studies have pointed out that there are gender differences in the host’s immune response to the hepatitis B vaccine, with women typically exhibiting a stronger response. This may be because estrogen stimulates monocytes to release IL-10, thereby promoting the secretion of IgG and IgM by B cells; conversely, testosterone inhibits the production of IgG/IgM and suppresses IL-6 derived from monocytes [32]. No significant effects of age or sex were observed in this study, which may be related to factors such as the narrow age range of the enrolled population and the limited sample size; these factors may have attenuated the influence of age and sex on the immune response. Additionally, some studies have indicated that vaccine immune responses may also be associated with factors such as obesity, smoking, and genetics [33].

Compared to the traditional three-dose regimen, the single-dose booster strategy offers significant advantages in settings such as universities, characterized by high population density and mobility. Although studies confirm superior immunogenicity with the three-dose schedule, actual coverage is often constrained by procedural complexity and low adherence. Unlike previous studies predominantly focused on booster immunization in general populations, this research represents the first systematic evaluation of the short-term immunogenicity of a single 60 μg vaccine dose in the specific cohort of Chinese university students. It pioneers the investigation of short-term antibody dynamics following 60 μg hepatitis B vaccine, thereby providing evidence for further catch-up vaccination and addressing a gap in the field.

However, this study has several important limitations. First, the relatively short follow-up period may lead to a simplified interpretation of antibody kinetics. Antibody decline typically follows a biphasic pattern, characterized by a rapid initial drop followed by a slower, stable plateau. Given our short follow-up period, our linear model may not fully and accurately reflect this process. Consequently, the linear model likely overestimates the initial decay rate of antibodies that are still in the “peak” phase, while potentially underestimating long-term persistence. This suggests that our 32.8 month prediction should be viewed as a conservative average estimate rather than a precise biological timeline. Accordingly, long-term follow-up studies with extended observation periods are warranted to validate the model-derived estimates and to refine the optimal timing for booster administration. Second, because this was a single-arm intervention study, no direct comparison with the standard vaccination schedule was made, making it impossible to directly compare efficacy. Therefore, although our study demonstrated the rapid development of serological protection, it was not possible to definitively assess the potential advantages or disadvantages of this single-dose regimen compared to the standard approach in terms of the long-term persistence of the immune response; consequently, future randomized controlled trials with parallel groups could be conducted. Finally, we did not systematically collect covariates that may influence immune responses, such as detailed vaccination history, smoking status, or genetic markers, which limits our ability to conduct a more nuanced analysis of interindividual variability.

## 5. Conclusions

This study reveals that although the prevalence of hepatitis B surface antigen positivity among Chinese university students is low, a substantial number of susceptible individuals exist within this cohort. A single 60 μg booster dose of hepatitis B vaccine can induce a rapid immune response in this population, with modeling predicting an average duration of protection reaching 2–3 years. Therefore, we recommend incorporating hepatitis B serological screening into university entrance medical examinations. Susceptible individuals should receive a single high-dose vaccine booster, followed by further assessment 2–3 years post-vaccination to determine the need for additional hepatitis B vaccine. This approach will establish an efficient campus hepatitis B prevention and control system.

## Figures and Tables

**Table 1:** Distribution of hepatitis B serological markers among screened students.

Serological Profile	*n* (%)
All markers negative	1883 (63.02%)
Anti-HBs^+^ only	1071 (35.84%)
Anti-HBs^+^ + Anti-HBc^+^	11 (0.37%)
Anti-HBc^+^ only	8 (0.27%)
HBsAg^+^ + Anti-HBe^+^ + Anti-HBc^+^	4 (0.13%)
HBsAg^+^ + Anti-HBc^+^	3 (0.10%)
HBsAg^+^ + HBeAg^+^ + Anti-HBc^+^	3 (0.10%)
Anti-HBe^+^ + Anti-HBc^+^	2 (0.07%)
Anti-HBe^+^ only	1 (0.03%)
Anti-HBs^+^ + Anti-HBe^+^	1 (0.04%)
Anti-HBs^+^ + Anti-HBe^+^ + Anti-HBc^+^	1 (0.05%)
Total	2988 (100%)

**Table 2:** The basic information of participants.

Charecteries	Value
Age	18.98 ± 0.83
Gender	
Male	61 (38.13%)
Female	99 (61.88%)
University	
A	52 (32.50%)
B	54 (33.75%)
C	54 (33.76%)

**Table 3:** SPR and GMC of anti-HBs after a single 60 μg hepatitis B vaccine booster.

Time Post-Vaccination	University A (*N* = 52)	University B (*N* = 54)	University C (*N* = 54)
Month 1			
SPR, % (*n*/*N*; 95% CI)	98.08 (51/52; 89.74–99.95)	87.04 (47/54; 75.10–94.63)	85.19 (46/54; 72.88–93.38)
GMC, mIU/mL (95% CI)	386.87 (256.10–584.41)	428.98 (224.19–820.83)	417.86 (201.80–865.27)
Month 2			
SPR, % (*n*/*N*; 95% CI)	100.00 (52/52; 93.15–100.00)	92.59 (50/54; 82.11–97.94)	92.59 (50/54; 82.11–97.94)
GMC, mIU/mL (95% CI)	475.05 (316.44–713.14)	373.94 (207.52–673.82)	396.61 (210.48–747.34)
Month 3/4/5 *			
SPR, % (*n*/*N*; 95% CI)	100.00 (52/52; 93.15–100.00)	90.74 (49/54; 79.70–96.92)	94.44 (51/54; 84.61–98.84)
GMC, mIU/mL (95% CI)	398.20 (258.79–612.71)	280.01 (161.94–484.14)	230.49 (131.02–405.46)

Note: * The follow-up visits at this time interval were conducted at different months: University A at Month 3, University B at Month 4, and University C at Month 5.

**Table 4:** Parameter estimates and standard errors of the model.

Component	Predictors	Estimate	Std. Error	*p*
Fixed effects	Intercept	2.64	0.07	<0.001
	Time (Month)	−0.05	0.01	<0.001
Random effects	*δ*_1_^2^	0.82		
	*δ*_2_^2^	0.02		
	*δ* ^2^	0.06		
Model fit indices	AIC	746.9		
	BIC	771.9		
	ICC	0.93		
	Marginal R^2^/Conditional R^2^	0.01/0.93		

Note: *δ*_1_^2^ represents the random intercept, *δ*_2_^2^ represents the random slope, and *δ*^2^ represents the residuals.

## Data Availability

Data are contained within the article.
